# Maximum Power Point Tracking-Based Model Predictive Control for Photovoltaic Systems: Investigation and New Perspective

**DOI:** 10.3390/s22083069

**Published:** 2022-04-16

**Authors:** Mostafa Ahmed, Ibrahim Harbi, Ralph Kennel, José Rodríguez, Mohamed Abdelrahem

**Affiliations:** 1 Chair of High-Power Converter Systems (HLU), Technical University of Munich (TUM), 80333 Munich, Germany; ibrahim.harbi@tum.de (I.H.); ralph.kennel@tum.de (R.K.); mohamed.abdelrahem@tum.de (M.A.); 2 Electrical Engineering Department, Faculty of Engineering, Assiut University, Assiut 71516, Egypt; 3 Electrical Engineering Department, Faculty of Engineering, Menoufia University, Shebin El-Koum 32511, Egypt; 4 Faculty of Engineering, Universidad San Sebastian, Santiago 8370146, Chile; jose.rodriguezp@uss.cl

**Keywords:** PV systems, MPPT, review, MPC, sensor reduction, fixed switching frequency, direct MPPT

## Abstract

In this paper, a comparative review for maximum power point tracking (MPPT) techniques based on model predictive control (MPC) is presented in the first part. Generally, the implementation methods of MPPT-based MPC can be categorized into the fixed switching technique and the variable switching one. On one side, the fixed switching method uses a digital observer for the photovoltaic (PV) model to predict the optimal control parameter (voltage or current). Later, this parameter is compared with the measured value, and a proportional–integral (PI) controller is employed to get the duty cycle command. On the other side, the variable switching algorithm relies on the discrete-time model of the utilized converter to generate the switching signal without the need for modulators. In this regard, new perspectives are inspired by the MPC technique to implement both methods (fixed and variable switching), where a simple procedure is used to eliminate the PI controller in the fixed switching method. Furthermore, a direct realization technique for the variable switching method is suggested, in which the discretization of the converter’s model is not required. This, in turn, simplifies the application of MPPT-based MPC to other converters. Furthermore, a reduced sensor count is accomplished. All conventional and proposed methods are compared using experimental results under different static and dynamic operating conditions.

## 1. Introduction

### 1.1. Toward Renewable Energy (PV Energy)

Currently, the utilization of renewable energy sources is increasing tremendously [1]. The issues of global warming, pollution, and emissions related to conventional sources have pushed and accelerated this increase [2,3]. Different countries are encouraging the use of the renewable sources [4,5]. The photovoltaic (PV) source is getting more attention among these sources. Numerous factors have contributed to this interest in PV energy. To mention a few, the PV system has a simple structure, where no moving parts or noisy instruments exist in this framework. It can be implemented for stand-alone or grid-connected applications. PV systems are commonly used for household purposes, where they can be installed on roofs of buildings [5]. The PV systems are executed under various structures. However, the classical configurations are the two-stage and single-stage topologies. The two-stage PV system is composed of a direct current–direct current (DC-DC) converter (the first stage) followed by an AC-DC inversion stage (the second stage). However, in the single-stage topology, the DC-DC converter is removed. Each configuration has its own merits and demerits, where the two-stage system simplifies the overall control strategy. Furthermore, the DC-DC stage boosts the voltage to an appropriate level for grid connection. However, the increased number of stages affects the conversion efficiency of the system. This also reflects on the size and cost of the system. The single-stage system requires a PV array configuration to enable grid integration (to obtain high voltage at the DC link). The control methodology in this topology is complicated. However, the conversion efficiency is higher compared to the single one [6,7,8,9,10]. Moreover, maximum power point tracking (MPPT) is an essential function in any PV system [11,12,13,14]. The PV source has a nonlinear characteristic, where under uniform radiation conditions, the power-voltage (P-V) curve has a unique maximum power point (MPP) [15]. Consequently, the objective of the MPPT is to operate the system at this point or practically as near as possible from this point [6].

In the literature, different MPPT algorithms are implemented, and therefore, different classifications exist for MPPT [16,17,18,19]. However, they can be mainly sorted into conventional methods, intelligent techniques, and nature-inspired or meta-heuristic algorithms. The conventional methods include well-known approaches such as perturb and observe (P&O) and incremental conductance (INC) [20,21]. Furthermore, other techniques that depend on the PV model or an analytical procedure lie in this group. Intelligent techniques employ fuzzy logic controllers or artificial neural networks to capture the maximum power [22]. However, nature-inspired or meta-heuristic methods are intended for partial shading conditions, where the utilized algorithm searches for the global maximum of the P-V curve when the nonuniform distribution of radiation over the PV modules happens [23]. A popular example of these methods is particle swarm optimization [24,25]. Furthermore, more advanced techniques are employed for global peak tracking such as leader-based collective intelligence algorithm, memetic salp swarm algorithm, and memetic reinforcement learning scheme [26,27,28]. Mainly, such algorithms are developed to accelerate and enhance the searching scheme of the traditional methods. Recently, model predictive control (MPC) is applied for various control objectives [29]. However, a limited number of studies have discussed the MPC-based MPPT implementation. This encourages the authors to focus on this new branch and investigate its performance in the PV system. Furthermore, new approaches for simplifying the execution of MPPT-based MPC are presented.

### 1.2. Previous Works on MPPT Based MPC

Table 1 summarizes the works that have been done in the area of MPPT based on MPC. From the table, the discussed techniques can be categorized into two groups of fixed switching methods [30,31] (based on a digital observer (DO)) and the finite-set model predictive control (FS-MPC) [32,33,34,35,36,37,38], which has a variable switching frequency. The fixed switching method depends on representing the PV model by an equivalent voltage source and a resistor. Therefore, the predicted PV current corresponding to a specific voltage can be calculated based on this equivalent circuit. To achieve a fixed switching behavior, a proportional–integral (PI) controller is used to compare the measured voltage with the reference one. Then, the resulted duty command is applied to a pulse width modulation (PWM) stage. In the FS-MPC, the discrete-time model of the utilized converter is used to select the best switching state according to a cost function design, where the predicted control parameter (voltage or current) is compared with its reference value. Thus, the switching state corresponding to the minimum cost function is chosen for application. Normally, the reference of the FS-MPC loop comes from the P&O or INC method, as stated in Table 1 (remarks column). The implementation of the fixed switching algorithm needs two sensors for the PV current and voltage. However, the FS-MPC implementation requires an additional sensor for the voltage at the output capacitor (due to dependency of the prediction stage on the model of the converter). This is considered a drawback for low-power PV applications. It is worth mentioning that different converter topologies are investigated in the aforementioned studies, where commonly, the boost converter is utilized for implementation. Other topologies such as flyback and buck converters are also studied.

### 1.3. Sensors Reduction for FS-MPC Implementation

Table 2 illustrates the sensor reduction approaches for the FS-MPC technique. As mentioned previously, the FS-MPC method requires additional sensors in the prediction stage (normally at the output terminal). This may be the cause of less interest in the FS-MPC as an MPP tracker. Therefore, some attempts have been made to tackle this problem. These methods can be categorized into two sets of methodologies. The first approach uses the model of the utilized converter (ideal one or including losses) to estimate the variable instead of measuring it [39,40,41]. The other technique employs an observer for estimation and sensor reduction. An extended Kalman filter is applied in [42] to estimate the input PV current of the boost converter. Similarly, a Luenberger observer is used with the P&O method in [43] for sensor current elimination. The observer design is also dependent on the discrete-time model of the system [3].

In view of the above, the MPPT-based MPC provides an elegant performance, where high efficiency can be obtained [44]. Furthermore, it has a fast dynamic response [45]. However, dependency on the utilized converter and additional sensors for the prediction stage are the major drawbacks of this technique. Moreover, calculation of the cost function increases its computational time. Therefore, the present research is aiming at simplifying the implementation of the FS-MPC for MPPT and reducing the number of required sensors. Additionally, for the fixed switching method (digital observer), a simple control law is used to remove the PI controller. The main contributions of our work can be summed up as follows:

- A review for the MPPT based on MPC is provided. Both fixed switching and variable switching frequency techniques are addressed.
- Analysis and implementation procedures of the MPPT-based MPC are discussed in detail.
- A modified approach for implementing the FS-MPC is proposed, where the discrete-time model of the system is not required. No cost function evaluation is needed, and sensor reduction is realized.
- A simple approach is used to eliminate the PI controller for the digital observer method, which decreases the tuning efforts.
- Experimental evaluation of all methods (the conventional and proposed) at different operating conditions (static and dynamic atmospheric profiles).

The remainder of this article is organized as follows: Section 2 presents the mathematical model of the PV system under study, whereas the implementation methods for MPPT using MPC are described in Section 3. The proposed methods with sensor reduction are investigated in Section 4. The experimental results, comparison, and evaluation are given in Section 5. Suggestions, key points, and future scope are addressed in Section 6. At last, the paper is finalized in Section 7.

## 2. PV System Modeling

**Figure 1:**
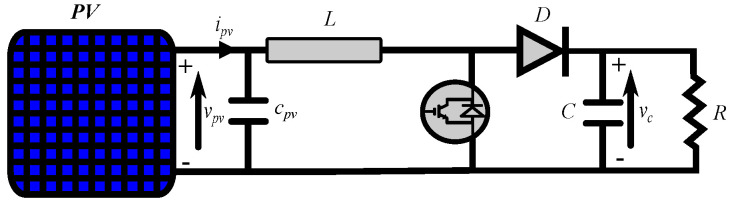
The configuration of the PV system with boost converter.

Different configurations are used for the PV system to assess the performance of the MPPT [46]. However, the most popular converter is the boost converter, especially when considering grid integration [3]. Therefore, the PV system under study utilizes the boost converter to interface the PV source with the load. Figure 1 simply shows this configuration.

### 2.1. Model of the PV Source

The PV source exhibits non-linear behavior. Therefore, numerous models have been utilized in the literature to describe such behavior. However, the most applied model is the single-diode one due to its simplicity and accuracy [47]. In this model, the current–voltage (I-V) behavior of the PV generator is given by [3,47]

(1) $$i_{pv}=i_{ph}-i_{o}\left[e^{\left(\frac{v_{pv}+i_{pv}R_{s}}{nN_{s}v_{th}}\right)}-1\right]-\frac{v_{pv}+i_{pv}R_{s}}{R_{sh}},$$

where $i_{ph}$ is the photovoltaic current, $i_{o}$ is the diode saturation current, *n* is the diode ideality factor, $R_{s}$ is the module series resistance, $R_{sh}$ is the module shunt resistance, $N_{s}$ is the number of cells in one module, $v_{th}$ is the thermal voltage, $i_{pv}$ is the terminal current, and $v_{pv}$ is the output voltage.

### 2.2. Model of the Boost Converter

The behavior of the boost converter is characterized by the actions of its switch, where two modes of operation are specified according to the switch state [48]. Hence, the model of the boost converter according to the state-space representation can be formulated as [3]

(2) $$\begin{array}{c} \dot{x}=Ax+Bu,\\ y=Cx+Du, \end{array}$$

where *x* = [$i_{pv}$$v_{c}$]${}^{T}$ is the state vector, *u* = $v_{pv}$ is the input PV voltage, and $y=v_{c}$ is the load output voltage. Furthermore, A, B, C, and D are the system matrices and are arranged as follows

(3) $$A=\left[\begin{array}{cc} 0 & -\frac{1-d}{L}\\ \frac{1-d}{C} & -\frac{1}{RC} \end{array}\right],\;B=\left[\begin{array}{c} \frac{1}{L}\\ 0 \end{array}\right],\;C=\left[\begin{array}{cc} 0 & 1 \end{array}\right],\;D=0,$$

where *L* is the boost inductance, *C* is the output capacitance, *R* is the load resistance, and *d* is the duty cycle of the boost converter.

## 3. MPC-Based MPPT

### 3.1. Fixed Switching Frequency MPPT-Based Digital Observer

**Figure 2:**
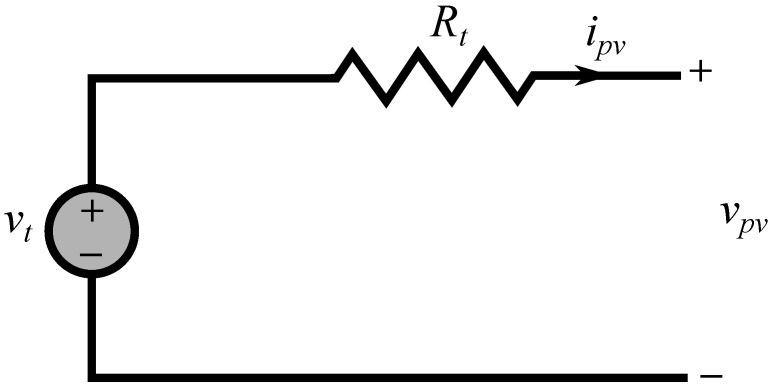
The simplified equivalent circuit of the PV generator.

The predictive fixed switching technique depends on the equivalent circuit of the PV generator. Figure 2 shows a simplified model of the PV source [31], which can be characterized by an equivalent voltage source connected in series with a total resistance. This model is called a digital observer, which can efficiently linearize the PV source model in the vicinity of the MPP [46]. Therefore, the design procedure of the fixed switching MPPT method can be performed following these steps [49,50]:

1. The predicted PV currents are calculated from

(4) $$i_{pv}{\left(k+1\right)}_{1,2}=i_{pv}\left(k\right)\pm \Delta i,$$

where $i_{pv}\left(k+1\right)$ is the PV current at the next sampling instant, $i_{pv}\left(k\right)$ is the PV current at the present sampling instant, and Δi is the step-size.

2. Consequently, the predicted voltages are calculated as

(5) $$v_{pv}{\left(k+1\right)}_{1,2}=v_{t}\left(k\right)-i_{pv}{\left(k+1\right)}_{1,2}\;R_{t}\left(k\right),$$

where $v_{t}\left(k\right)$ and $R_{t}\left(k\right)$ are the equivalent voltage and resistance of the PV source, respectively.

3. The equivalent resistance and voltage of the PV source are computed as follows

(6) $$R_{t}\left(k\right)=-\frac{v_{pv}\left(k-1\right)-v_{pv}\left(k\right)}{i_{pv}\left(k-1\right)-i_{pv}\left(k\right)},$$

(7) $$v_{t}\left(k\right)=v_{pv}\left(k\right)+i_{pv}\left(k\right)R_{t}\left(k\right),$$

where $v_{pv}\left(k-1\right)$ and $i_{pv}\left(k-1\right)$ are the PV voltage and current at the previous sampling instant.

4. The cost function for assessment and selection is based on the predicted PV power as

(8) $$p_{pv}{\left(k+1\right)}_{1,2}=v_{pv}{\left(k+1\right)}_{1,2}\;i_{pv}{\left(k+1\right)}_{1,2}.$$

The predicted power is compared with the present PV power. Therefore, the cost function is finalized as

(9) $$g_{1,2}=p_{pv}{\left(k+1\right)}_{1,2}-p_{pv}\left(k\right).$$

According to the cost function, the PV voltage corresponding to the higher power (between $p_{pv}{\left(k+1\right)}_{1}$ and $p_{pv}{\left(k+1\right)}_{2}$) will be chosen. Conventionally, this voltage is compared with the actual one, and the duty cycle is obtained using a PI controller. However, we suggest the following procedure, which implies elimination of the PI controller. An adjustable step is used to get the duty cycle command, where the function of this step is to minimize the difference between the optimal voltage and the actual value. Therefore, the design of the tunable step is obtained from

(10) Δd=c|Δv|,

where *c* is an adjustable factor, and Δv is the difference between the optimal voltage and the actual value.

**Figure 3:**
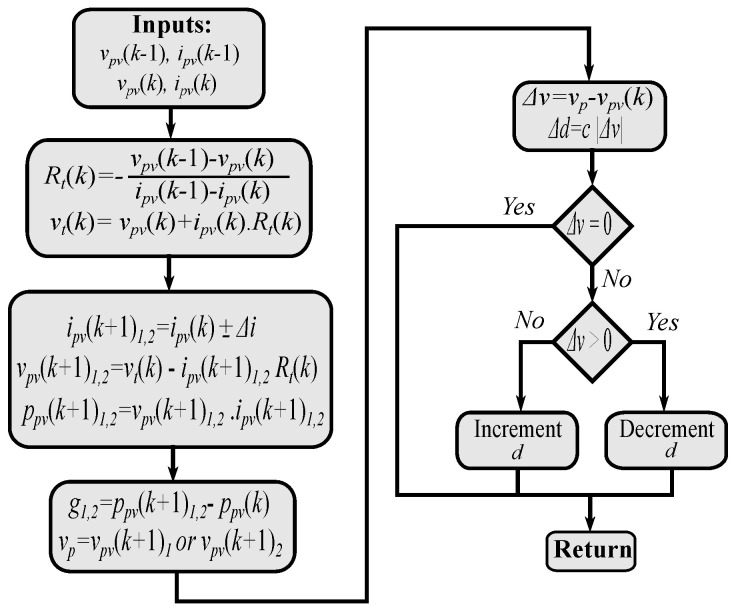
Predictive fixed switching frequency MPPT based digital observer without PI controller.

Figure 3 shows the working procedure of the predictive fixed switching MPPT without a PI controller. Firstly, the equivalent values of the PV source based on the present and previous measurements are calculated. Then, the predicted currents are computed utilizing a fixed step for the current. Following, the predicted voltages are obtained, which enable the calculation of the predicted values of the power. According the cost function design, the best voltage is selected. Finally, the duty cycle command is obtained using an adjustable step for the distance between the actual and the optimal voltage.

### 3.2. Variable Switching Frequency MPPT-Based FS-MPC

To implement the FS-MPC, the discrete-time model of the system is required. Therefore, and with reference to the model of the boost converter (Equation (3)), one can derive the following [37,51,52]

(11) $$i_{pv}\left(k+1\right)=i_{pv}\left(k\right)+\frac{T_{s}}{L}\left[v_{pv}\left(k\right)-v_{c}\left(k\right)\right],$$

(12) $$v_{c}\left(k+1\right)=\left[1-\frac{T_{s}}{RC}\right]v_{c}\left(k\right)+\frac{T_{s}}{C}i_{pv}\left(k\right),$$

(13) $$i_{pv}\left(k+1\right)=i_{pv}\left(k\right)+\frac{T_{s}}{L}v_{pv}\left(k\right),$$

(14) $$v_{c}\left(k+1\right)=\left[1-\frac{T_{s}}{RC}\right]v_{c}\left(k\right),$$

where Equations (11) and (12) represent the OFF state, while the other two equations represent the ON state. Furthermore, $T_{s}$ is the sampling period, k+1 is the predicted sampling instant, and *k* is the current one. Simply, putting the previous discrete-time model in a matrix form gives

(15) $$A_{d}=\left[\begin{array}{cc} 1 & -\frac{T_{s}\left(1-d\right)}{L}\\ \frac{Ts(1-d)}{C} & 1-\frac{T_{s}}{RC} \end{array}\right],\;B_{d}=\left[\begin{array}{c} \frac{T_{s}}{L}\\ 0 \end{array}\right],\;C_{d}=\left[\begin{array}{cc} 0 & 1 \end{array}\right],\;D_{d}=0,$$

where $A_{d}$, $B_{d}$, $C_{d}$, and $D_{d}$ are the discretized form of the system matrices.

The MPPT using FS-MPC can be carried out based on the current or voltage design. Therefore, for the current design, the cost function is

(16) $$g_{{i|}_{s=0,1}}=\left|i_{pv_{s=0,1}}\left(k+1\right)-i_{r}\right|,$$

where $i_{r}$ is the reference current. However, to accomplish the voltage design, the predicted PV voltages should be evaluated as follows

(17) $$v_{pv}\left(k+1\right)=\left[1-d\right]v_{c}\left(k+1\right).$$

Then, the cost function for optimal switching state selection is defined as

(18) $$g_{{v|}_{s=0,1}}=\left|v_{pv_{s=0,1}}\left(k+1\right)-v_{r}\right|,$$

where $v_{r}$ is the reference voltage. The cost function design can include both current and voltage. However, a weighting factor is required, and the final design is formulated as [46]

(19) $$g_{{i,v|}_{s=0,1}}=|v_{pv_{s=0,1}}\left(k+1\right)-v_{r}\left|+\lambda \right|i_{pv_{s=0,1}}\left(k+1\right)-i_{r}|.$$

**Figure 4:**
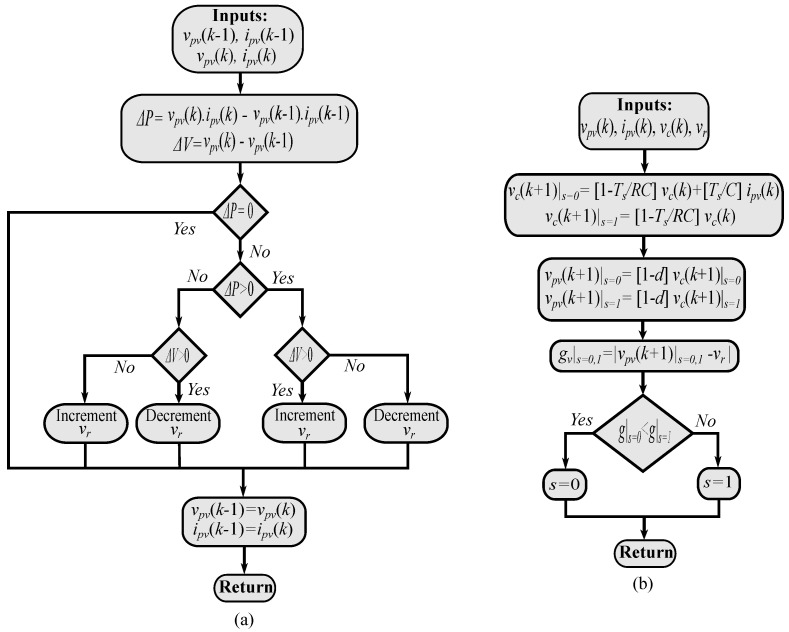
MPPT-based FS-MPC: (**a**) Reference voltage calculation using P&O method. (**b**) Optimal switching state selection-based FS-MPC.

The final algorithm for the FS-MPC is shown in Figure 4, where the P&O method is used to generate the reference voltage for the FS-MPC loop (the voltage design is chosen here for implementation). Finally, the optimal state is selected within the prediction stage of the FS-MPC according to the cost function design.

Similar to the conventional methods (P&O and INC), the fixed switching MPPT uses two sensors for the PV voltage and current to accomplish the MPPT operation. For the case of FS-MPC, an additional sensor for the output capacitor voltage is required during the prediction stage. Therefore, in total, the MPPT-based FS-MPC needs three sensors to track the maximum power. However, no modulator is required as the case of the fixed switching technique.

## 4. The Proposed Direct MPPT Techniques

The first proposed methodology depends on the direct control method, in which the duty cycle is perturbed directly in the control technique [53]. In the suggested algorithm, the duty cycle is replaced with the switching state as the case of the FS-MPC. However, the switching state is predicted directly after the measurement stage without the need for a discrete-time model of the converter. Therefore, the evaluation of cost function is also eliminated. This technique is firstly suggested in [3] (previous publication from the authors). However, only simulation results are provided there.

The second developed approach is based on the indirect P&O method. Conventionally, and after reference calculation, this reference is sent to the PI controller to get the duty cycle [54]. However, according to the FS-MPC, the PI controller can be eliminated, and a cost function is used to obtain the optimal switching state [46]. Therefore, in the proposed algorithm, a similar approach is utilized. As there are only two states of the boost converter, the authors suggest selecting the best switching state after reference calculation and without the need for the cost function evaluation. It is important to notice that the cost function is mandatory for systems where several cases exist to allocate the best switching state. To be specific, and for example, the two-level inverter has eight switching vectors, and the cost function is required to select among them [55]. However, in our case, the DC-DC converter has only two states. This simplifies the mission and therefore the procedure for obtaining the switching state can be implemented as follows:

- The difference between the reference voltage and the measured one is calculated as
  (20) $$v_{d}=v_{r}-v_{pv}.$$

- The switching actions are determined from
  (21) $$s=\left\{\begin{array}{cc} 0, & \mathrm{if}\;v_{d}>0,\\ 1, & \mathrm{if}\;v_{d}<0. \end{array}\right.$$

**Figure 5:**
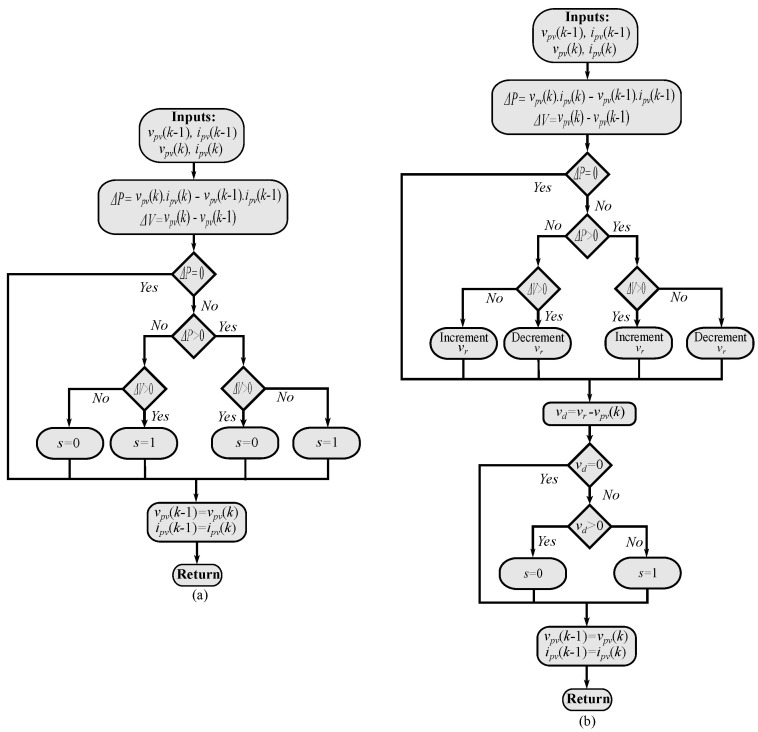
The proposed MPPT techniques: (**a**) MPPT-based direct P&O. (**b**) MPPT-based indirect P&O.

Figure 5 shows the two suggested techniques for MPPT. It can be noted that only two sensors are used in the proposed scheme just like the conventional methods and with fewer sensors in comparison with the FS-MPC approach. Furthermore, the discrete-time model of the system is not required, where a simple approach is utilized to select the optimal switching state. Moreover, the evaluation of the cost function is eliminated, which simplifies the implementation and reduces the computational burden. Due to similarity with the FS-MPC principle, the authors recall the proposed method as direct maximum power point tracking (DMPPT).

## 5. Experimental Results

### 5.1. Test Bench Description

**Figure 6:**
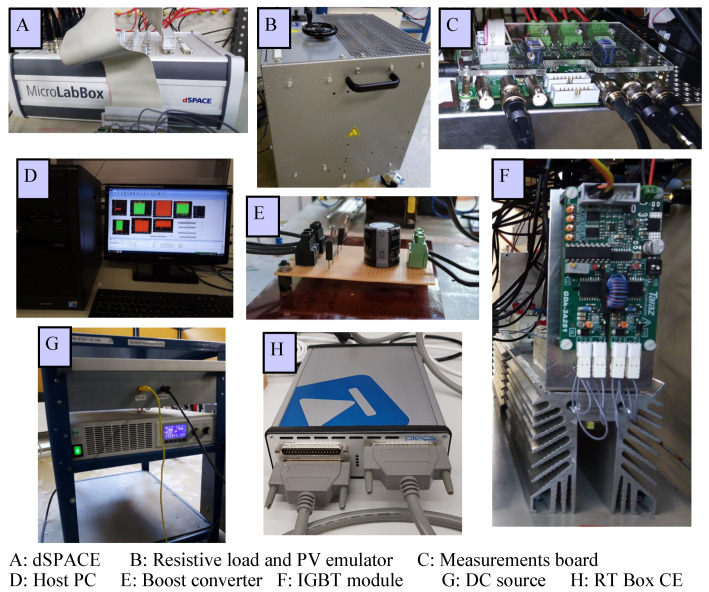
The experimental configuration of the PV system and HIL.

The system under consideration consists of a PV emulator, boost converter, and resistive load. The PV emulator is constructed using a DC source and a group of parallel-connected resistors [56]. Firstly, the DC source is connected with one resistor of this group to emulate the P-V curve at a certain power value. Then, and after a certain interval, another resistor is connected in parallel to the first one to emulate a sudden increase in the power. At last, the added resistor is removed to simulate an abrupt decrease in the output power of the emulator. The utilized real-time controller is the dSPACE MicroLabBox. The control algorithm is developed using Matlab software, and hence, the generated switching state is applied to the power switch of the boost converter. To assess the performance of the PV system under dynamic weather conditions, hardware-in-the-loop (HIL) is used to build the PV system under study. The HIL (RT Box CE) enables the implementation and testing of the system under different operating conditions. Figure 6 shows the experimental arrangement of the set-up, while the parameters of the PV system are summarized in Table 3.

### 5.2. Evaluation and Discussions

The performance of all methods is firstly investigated under step changes of the input PV power. The comparison includes the fixed switching method based digital observer (DO), the FS-MPC technique, the first developed MPPT based direct P&O (DMPPT1), and the second proposed approach based indirect P&O (DMPPT2).

**Figure 7:**
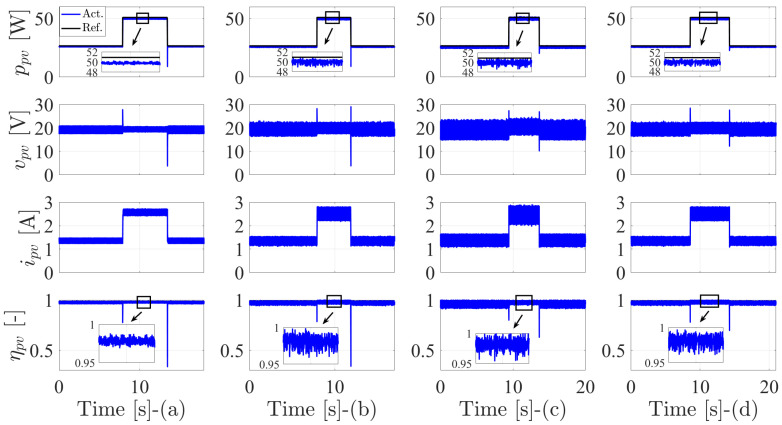
The performance of the MPPT techniques under step response of the PV power: (**a**) Fixed switching MPPT-based digital observer. (**b**) MPPT-based FS-MPC. (**c**) The first proposed DMPPT technique-based direct P&O. (**d**) The second proposed DMPPT technique-based indirect P&O.

Figure 7 shows the behavior of all previously mentioned methods, where the PV power ($p_{pv}$), PV voltage ($v_{pv}$), PV current ($i_{pv}$), and instantaneous efficiency ($\eta_{pv}$) are presented. The PV power has the lowest ripple content at steady state with the MPPT-based DO. This is observed in the waveform of the instantaneous efficiency. Thanks to the modulator and fixed switching behavior of this method, the FS-MPC method and DMPPT2 have a very similar behavior. However, the ripple content of the PV power with the proposed DMPPT2 is a little bit enhanced, which can be seen in the oscillations of the instantaneous efficiency. The first proposed DMPPT1 has the highest ripple content among all studied methods, which is clearly observable in different waveforms of voltage, current, and efficiency.

Table 4 summarizes the behavior of the studied methods concerning the average efficiency and tracking speed. The average efficiency is calculated as specified in [57]. The efficiency of the MPPT-based DO is the highest among all methods. Furthermore, the proposed DMPPT2 comes in the second place. The MPPT-based FS-MPC comes at third position, and finally, the proposed DMPPT1 with approximately 0.5% difference between this method and the fixed switching one. Concerning the tracking speed, the fixed switching method has a remarkable slow transient response at a step change of the PV power in comparison to other methods. The proposed DMPPT2 and FS-MPC techniques are the fastest. However, the DMPPT1 has a slow tracking speed by one sample in comparison to DMPPT2 and the FS-MPC approach thanks to the MPC principle, which provides fast dynamics.

Further investigation is provided by comparing the average switching frequency and the computational load of all methods. This is given in Table 5, where the MPPT-based DO has a significantly higher computational burden in comparison to other methods. This time is consumed in the calculation of the digital observer of the PV source and the prediction of the optimal voltage. Following, and concerning computational load, the FS-MPC comes in second place, where the prediction stage and cost function evaluations require an additional interval. However, the proposed DMPPT1 and DMPPT2 have a very similar computational time that is considered the lowest among all methods, where no dependency on the model of the PV system is involved. Furthermore, the cost function calculation is not required in these methods. The switching frequency for the fixed method is chosen to be comparable to the variable switching methods to have a fair comparison. The FS-MPC and DMPPT2 have approximately the same average switching frequency. However, the average switching frequency of DMPPT1 is the lowest, which explains its modest steady-state performance.

To evaluate the performance of the MPPT methods at dynamic conditions as recommended by the European efficiency test (EN50530) [58], a similar dynamic waveform which is a triangular function is used. This function is built in the utilized HIL system and has the rising and falling ramp manner. Therefore, it is used in this study to assess the performance of the MPPT techniques. The utilized PV module in the HIL system is KC200GT [3,6].

**Figure 8:**
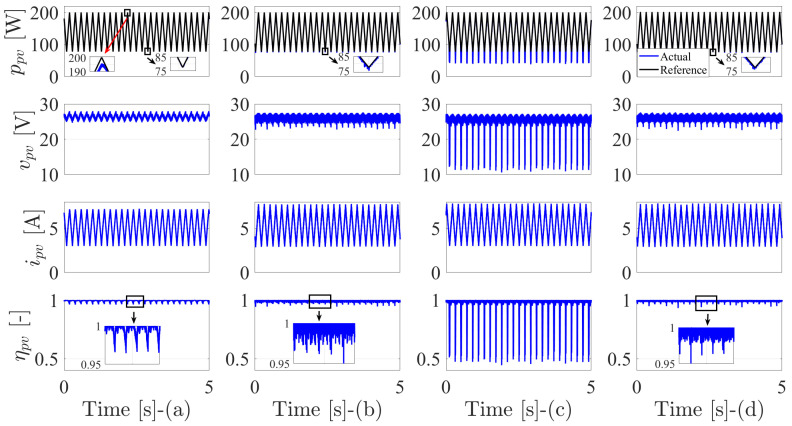
The performance of the MPPT techniques under dynamic weather conditions: (**a**) Fixed switching MPPT-based digital observer. (**b**) MPPT-based FS-MPC. (**c**) The first proposed DMPPT technique-based direct P&O. (**d**) The second proposed DMPPT technique-based indirect P&O.

Figure 8 shows the response of the MPPT algorithms when the triangular radiation profile is applied for the input of the PV source. As the PV power has a proportional relation with the radiation, the extracted (produced) power is behaving similarly to the radiation profile (triangular). The PV power with the MPPT-based DO is exhibiting a drift behavior as clarified in the zoomed part of the upper peak of the triangular waveform. It is obvious that the drift occurs to the right of the MPP, where the PV voltage increases and the PV current decreases at this side of operation. This is clearly notable in the waveform of the voltage and current. Therefore, and corresponding to the drift occurrence, the instantaneous efficiency drops. However, the behavior of the PV power at the lower peak of the triangular waveform is very sufficient with the DO method. In contrast, the drift phenomenon occurs at the left side of the MPP for the other methods and in conjunction with the lower peak of the PV power. It should be mentioned that the drift occurrence is uncontrollable [57]. Further details about the drift or divergence of MPPT techniques at fast-changing atmospheric conditions can be found in [6,57,59]. The MPPT-based FS-MPC and DMPPT2 have similar behavior when considering the ramp variation of the PV power, where the drift occurrence for those two methods is quite similar, as can be seen at the lower peak of the PV power. Corresponding to this divergence, there is a periodic decrease in the PV voltage (the drift occurs at the left side of MPP). However, the proposed DMPPT2 exhibits a slightly enhanced waveform, which can be observed in the zoomed part of the PV power. The proposed DMPPT1 has the worst behavior at dynamic weather variation, where the drift occurrence is very notable in the PV power and voltage waveforms. This, in turn, causes a significant drop in the instantaneous efficiency. Furthermore, and as mentioned previously, the low average switching frequency of this method contributes to the PV power and voltage oscillations.

**Figure 9:**
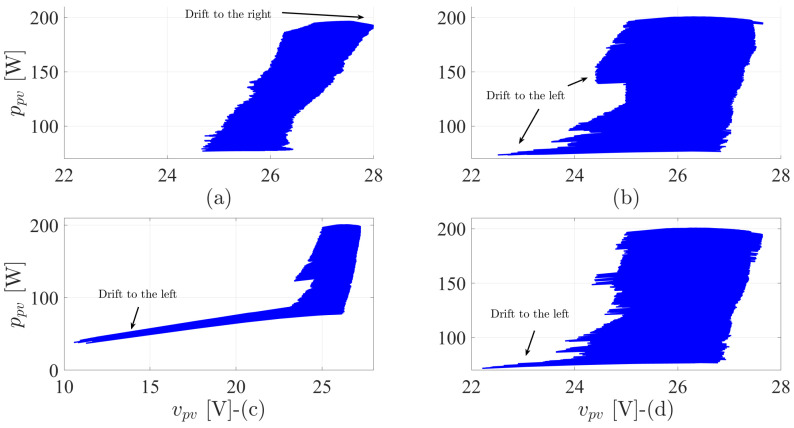
The power–voltage curves of the MPPT techniques under dynamic weather conditions: (**a**) Fixed switching MPPT-based digital observer. (**b**) MPPT-based FS-MPC. (**c**) The first proposed DMPPT technique-based direct P&O. (**d**) The second proposed DMPPT technique-based indirect P&O.

To further clarify the drift phenomenon, the power-voltage variations in dynamic weather conditions are investigated in Figure 9. The fixed switching technique is diverging to the right of MPP, as clarified in the voltage values. Other methods are drifting to the left of the MPP. The FS-MPC and DMPPT2 have similar divergent behavior. However, the performance of the proposed DMPPT2 is improved at high power values. The drift behavior of the DMPPT1 is bad when compared to the FS-MPC and DMMPT2, especially at low power values. However, at high power conditions, it is similar to the FS-MPC technique.

As a summary, Table 6 provides the efficiency of the MPPT methods and average switching frequency at dynamic radiation variation. The proposed DMPPT2 has the highest efficiency among all studied methods. However, the proposed DMPPT1 efficiency is the lowest. Furthermore, the average switching frequency at dynamic conditions is similar for the variable switching methods.

Moreover, Table 7 gives a comparative summary of the behavior of MPPT algorithms when considering the static and dynamic conditions studied previously. Different aspects have been considered in this comparison. However, to sum up, the FS-MPC uses more sensors in comparison to other methods. The steady-state behavior of the fixed switching method is the best among all methods. However, it has a slow transient behavior. Furthermore, its computational load is the highest. All methods suffer from divergence in dynamic weather conditions. However, this drift is very noticeable with the DMPPT1 method. The proposed DMPPT2 technique has the most adequate performance among all methods considering the efficiency, drift, transient behavior, and computational burden.

## 6. Recommendation and Future Scope

In the present study, a review of the MPPT techniques based on the MPC approach has been presented. In this framework, new perspectives have been proposed to simplify the implementation procedure, which is inspired by the MPC algorithm. However, the research in this direction can be further enhanced by addressing the following suggestions and recommendations:

- Sensor reductions are highly advisable for MPPT-based MPC, especially for the FS-MPC technique. This of course reduces the implementation cost for low-power PV applications. Furthermore, the reliability of the system will be enhanced in case of sensor failure. Therefore, the system can operate with a minimum number of sensors. Moreover, such sensor reduction approaches can effectively assist the system as backup control.
- In the same context, several analytical and observer-based methods are recommended to accomplish the sensor reduction schemes.
- Simplifications are also encouraged to be applied for the FS-MPC to suit the low-cost implementation platforms.
- Multi-objective control can and should be executed for the FS-MPC technique. This approach adds flexibility to the control problem formulation, which can be advantageous in several applications (not only MPPT). Moreover, new constraints can be easily added to follow the continuous development of the control objectives and grid-codes modification (in case of grid-connected applications).
- Integration of the optimization techniques into the MPC method to address the partial shading conditions and solve the problem of power loss under such circumstances. However, this may come at the cost of higher computational power.
- The performance of the MPPT under fast-changing atmospheric conditions should be carefully investigated. Furthermore, the drift or divergence of the MPPT method is to be analyzed and solved.
- Fixed switching frequency implementation is preferred in some applications such as grid-connected systems (to simplify filter design). Therefore, specific requirements of the system should be taken into consideration.

## 7. Conclusions

In this article, an overview of the MPPT-based MPC is proposed, where the fixed switching method and the variable switching one are addressed. The PI controller for the fixed MPPT-based DO method is eliminated, and an adaptive step-size is used to minimize the difference between the predicted PV voltage and the actual value. This, in turn, decreases the tuning efforts and simplifies the execution procedure. Based on the FS-MPC principle, two approaches are developed for MPPT named direct MPPT (DMPPT). The proposed methods exploit the MPC principle to select the optimal switching state without the need for cost function evaluations, which greatly decreases the computational burden and simplifies the implementation of the FS-MPC procedure for different converter topologies, as there is no need to derive the discrete-time model of the utilized converter. The FS-MPC and direct methods give tracking speed, which is almost three times faster when compared to the fixed switching technique under step change condition. More specifically, the proposed DMPPT2 gives higher efficiency and fast dynamics in comparison with other methods, where an average efficiency of 97.55% under static experimental tests is achieved. Additionally, and using dynamic HIL tests, an efficiency of 99.67% is obtained. Furthermore, a smaller number of sensors is required in comparison with the conventional FS-MPC due to the absence of a prediction stage, where only two voltage and current sensors (one voltage sensor saving compared to the FS-MPC) are utilized. However, still, all methods suffer from the drift phenomenon, especially the DMPPT1 technique. Simplification of the FS-MPC, sensor reduction techniques, and drift-avoidance are encouraged for future directions in this area of research.

## Figures and Tables

**Table 1:** Previous works on MPPT-based MPC.

Cited Reference	Converter Topology	Number of Sensors	Implementation	Remarks
[30]	Boost converter	2	Experimental	A digital observer for the PV model is used to predict the optimal voltage and achieve a fixed switching frequency. Furthermore, the duty cycle command is obtained by a PI controller.
[31]	Z-source inverter	2	Experimental	Similar to the above mentioned technique, i.e., [30].
[32]	Boost converter	3	Simulation	INC method is used to provide the reference current for the FS-MPC loop with two-step prediction.
[33]	Boost converter	4	Experimental	Modified P&O algorithm is implemented for reference generation with various voltage and current-based FS-MPC techniques.
[34]	Flyback converter	3	Simulation	FS-MPC is combined with the INC method to improve its transient behavior.
[35]	Buck converter	3	Experimental	Technique for dynamic atmospheric conditions is implemented, where a model for the PV source is combined with the FS-MPC to improve the system’s performance.
[36]	Boost converter	3	Experimental	Revised version of the P&O method with one-step prediction is executed and integrated with the FS-MPC approach.
[37]	Boost converter	3	Simulation	P&O method is utilized as a reference generator for the FS-MPC.
[38]	Boost converter	3	Simulation	INC method-based reference current tracking is combined with the FS-MPC algorithm.

**Table 2:** Sensor reduction approaches for MPPT-based FS-MPC.

Cited Reference	Converter Topology	Number of Sensors	Implementation	Remarks
[39]	Flyback converter	2	Experimental	INC is utilized to generate the reference for the FS-MPC. The current sensor is eliminated based on the MPC approach. Furthermore, a simple load observer is included.
[40]	Multilevel boost converter	2	Experimental	INC method is employed for the FS-MPC technique. Sensor reduction is accomplished using a simplified model for a multilevel converter.
[41]	High gain DC-DC converter	2	Experimental	INC method is used with the FS-MPC method. The output voltage sensor is removed using the voltage gain equation of the utilized converter.
[42]	Boost converter	2	Simulation	P&O method is used as a reference generator for the FS-MPC. The required sensors are reduced by employing an extended Kalman filter.

**Table 3:** The parameters of the experimental set-up.

Parameter	Value
Boost inductor (L)	8.5 mH
Output capacitance (C)	240μF
Power switch	IGBT-Module FF50R12RT4
Diode (D)	fast recovery diode BYW77PI200
Load (R)	30Ω
PV emulator resistors	15Ω/16.5Ω
Sampling time $\left(T_{s}\right)$	100μs

**Table 4:** Tracking speed and average efficiency of the studied MPPT methods.

Method	Tracking Speed	$\eta_{pv,avg}$ (%)
MPPT based DO	13 $T_{s}$	97.57
MPPT based FS-MPC	4 $T_{s}$	97.52
DMPPT1	5 $T_{s}$	97.10
DMPPT2	4 $T_{s}$	97.55

**Table 5:** Execution time and average switching frequency for the MPPT algorithms.

Method	Execution Time (μs)	Avg. $f_{s}$ (kHz)
MPPT-based DO	5.67	3.33 (fixed)
MPPT-based FS-MPC	5.25	3.67
DMPPT1	4.87	2.42
DMPPT2	4.90	3.66

**Table 6:** The average efficiency and average switching frequency for the MPPT algorithms at dynamic radiation conditions.

Method	$\eta_{pv,avg}$ (%)	Avg. $f_{s}$ (kHz)
MPPT-based DO	99.58	3.33 (fixed)
MPPT-based FS-MPC	99.66	2.41
DMPPT1	99.28	2.48
DMPPT2	99.67	2.46

**Table 7:** Comparative assessment of the MPPT techniques.

Parameter	MPPT-Based DO	MPPT-Based FS-MPC	DMPPT1	DMPPT2
Number of utilized sensors	2	3	2	2
Switching frequency	Fixed	Variable	Variable	Variable
Computation burden	Very High	High	Low	Low
Tracking speed	Slow	Very fast	Fast	Very fast
Cost function evaluation	Required	Required	Not required	Not required
Steady-state behavior	Excellent	Very good	Good	Very good
Dynamic behavior	Drift occurs	Drift occurs	Drift occurrence is significant	Drift occurs
System model’s dependency	Exists (DO)	Exists (discrete model)	No	No
Efficiency	Very high	Very high	High	Very High

## Data Availability

Not applicable.

## Abbreviations

MPPT	Maximum power point tracking
MPC	Model predictive control
PV	Photovoltaic
PI	Proportional–integral
MPP	Maximum power point
P&O	Perturb and observe
INC	Incremental conductance
PWM	Pulse width modulation
FS-MPC	Finite-set model predictive control
DC	Direct current
DO	Digital observer
**Symbols**	
$i_{ph}$	Photovoltaic current
*n*	Diode ideality factor
$i_{o}$	Diode saturation current
$R_{s}$	Module series resistance
$R_{sh}$	Module shunt resistance
$v_{th}$	Thermal voltage
$N_{s}$	Number of series cells in one module
$v_{pv}$	Output voltage of PV source
$i_{pv}$	Output current of PV source
**A, B, C, D**	System model matrices
$v_{c}$	Capacitor voltage
*L*	Boost inductance
*C*	Boost output capacitance
*R*	Load resistance
*d*	Duty cycle of the boost converter
$v_{t}$	Equivalent voltage of the PV source
$R_{t}$	Equivalent resistance of the PV source
*g*	Cost function
$T_{s}$	Sampling time
λ	Weighting factor
